# Supplementary material for: Transcriptome Analysis of Sweet Cherry (Prunus avium L.) Cultivar ‘Lapins’ upon Infection of Pseudomonas syringae pv. syringae
Source: Plants (Basel). 2023 Oct 29;12(21):3718. doi: 10.3390/plants12213718 (PMC10647540; doi:10.3390/plants12213718)
Supplement: Supplementary file 1 [file plants-12-03718-s001.zip › Cui_Supp.Figs_edited.pdf]

A

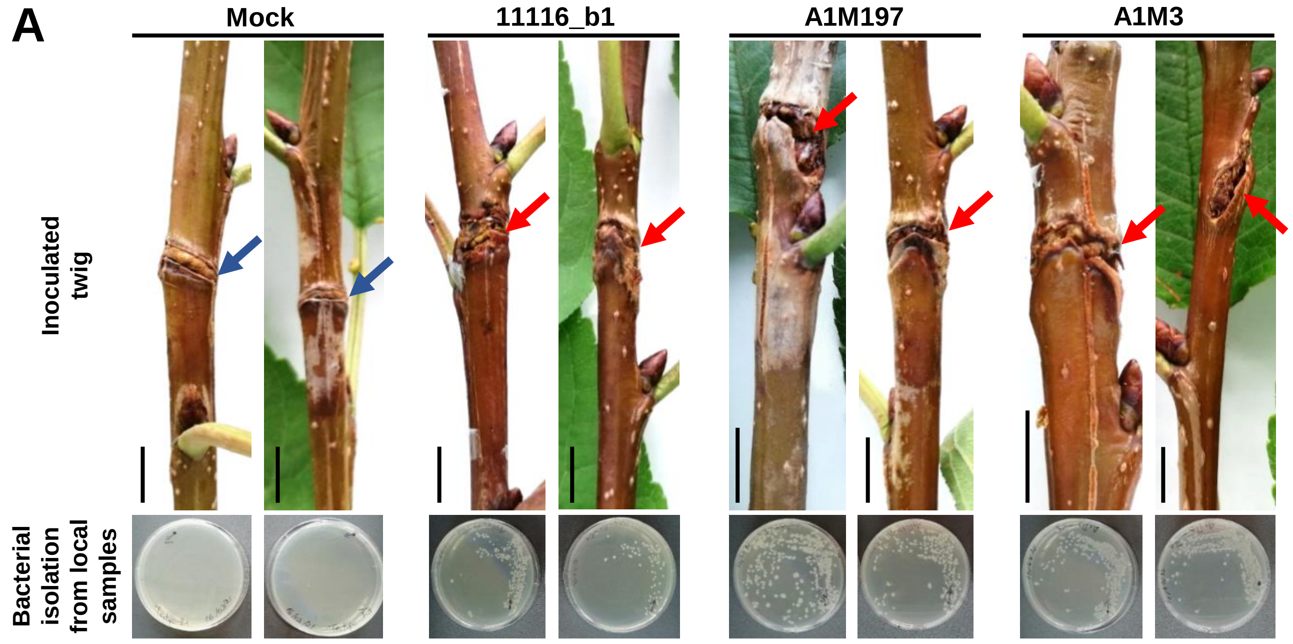

B

		Colonies isolated from local samples					
Pss strain inoculated in twig	Sample	UV fluorescence	Levan production	Oxidase production	Pectinolytic activity	Arginine hydrolase production	Tobacco Hyper-sensitivity
11116_b1	1	+	+	-	-	-	+
	2	+	+	-	-	-	+
A1M197	1	+	+	-	-	-	+
	2	+	+	-	-	-	+
A1M3	1	+	+	-	-	-	+
	2	+	+	-	-	-	+

C

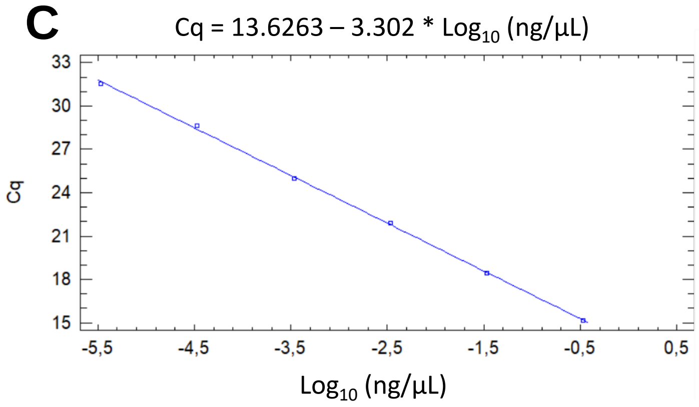

D

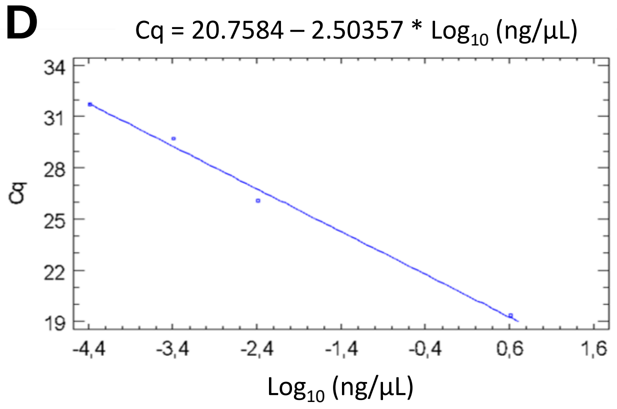

Supplementary Figure S1. Evaluation of Pss infection on sweet cherry and calibration curves to quantify Pss DNA in plant samples. (A) Upper panels: twigs from sweet cherry cv. Lapins were inoculated by wounding with three Pss strains, 11116_b1, A1M197, and A1M3, in a $2x10^8$ CFU/mL suspension. Sterile saline buffer was used as mock control. After 40 days, open wounds on twigs with Pss infection were observed (red arrows), whereas the mock control twigs showed closed wounds (blue arrows). Lower panels: viable bacteria were isolated from local samples inoculated with Pss. Scale bars, 1 cm. (B) Isolated colonies from two inoculated local samples per Pss strain were analyzed by UV fluorescence and LOPAT test. All colonies exhibited fluorescence under UV exposure, tested positive for levan production, negative for oxidase production, negative for pectinolytic activity in potato, negative for arginine hydrolase production, and triggered hypersensitive response in tobacco leaves. Two colonies per plant sample were evaluated. (C)-(D) Calibration curves for Pss and *Prunus avium* DNA quantification were obtained by detecting the glutathione-dependent formaldehyde-activating enzyme 1 gene in purified A1M3 strain DNA (C) and the protein SAR DEFICIENT 1 gene in DNAs purified from *Prunus Avium* twig (D). The equation between Cq and DNA concentration is shown. Cq, quantification cycle.

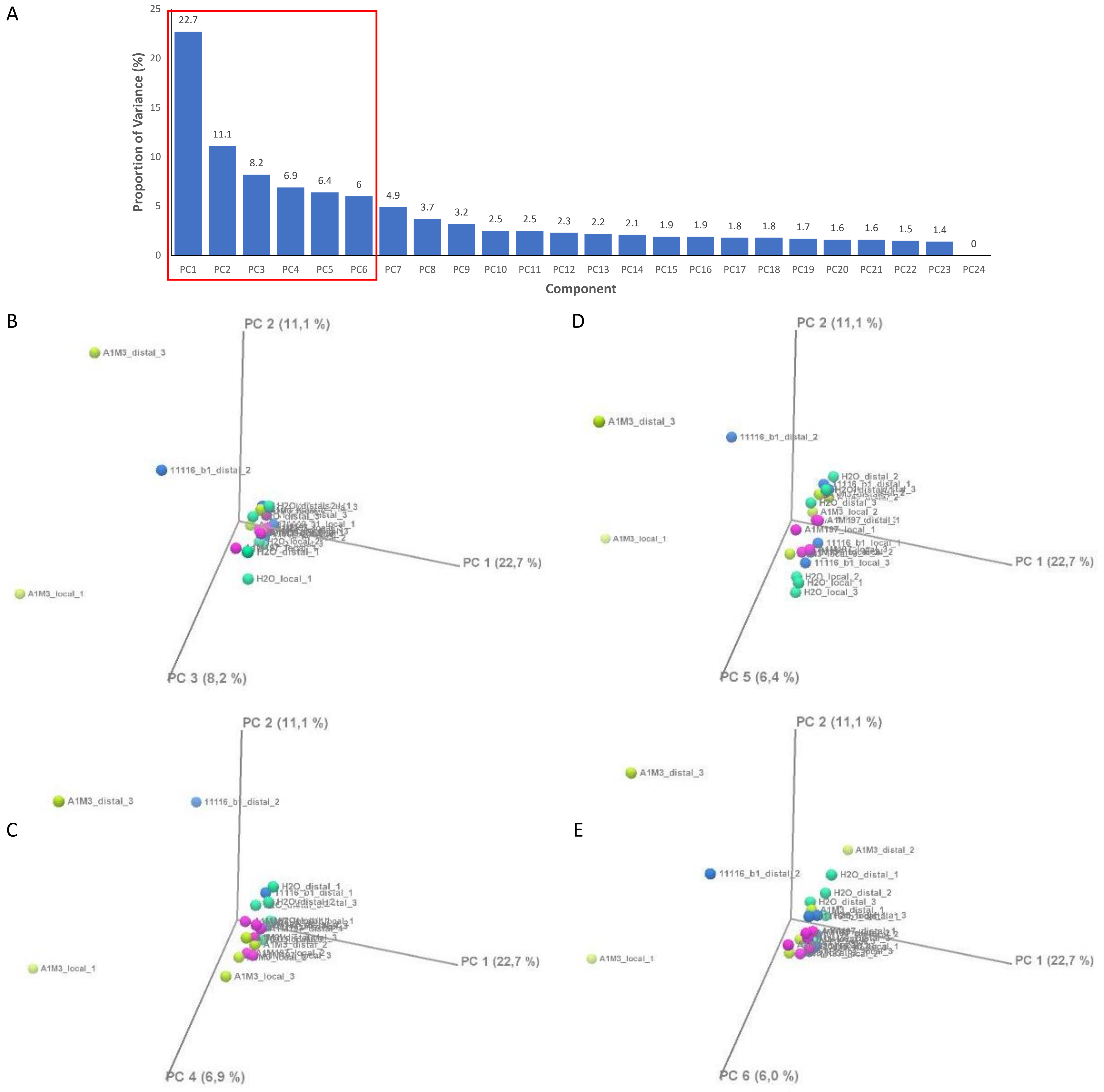

Supplementary Figure S2. Principal component (PC) analysis of the transcriptome datasets from the 24 samples of sweet cherry inoculated with Pss strains. (A) Proportions of variances. The red rectangle indicates the first six PCs, the sum of proportions of which exceeds 60%. (B)-(E) 3D scatter plots showing the first two PCs and one of the following PCs.

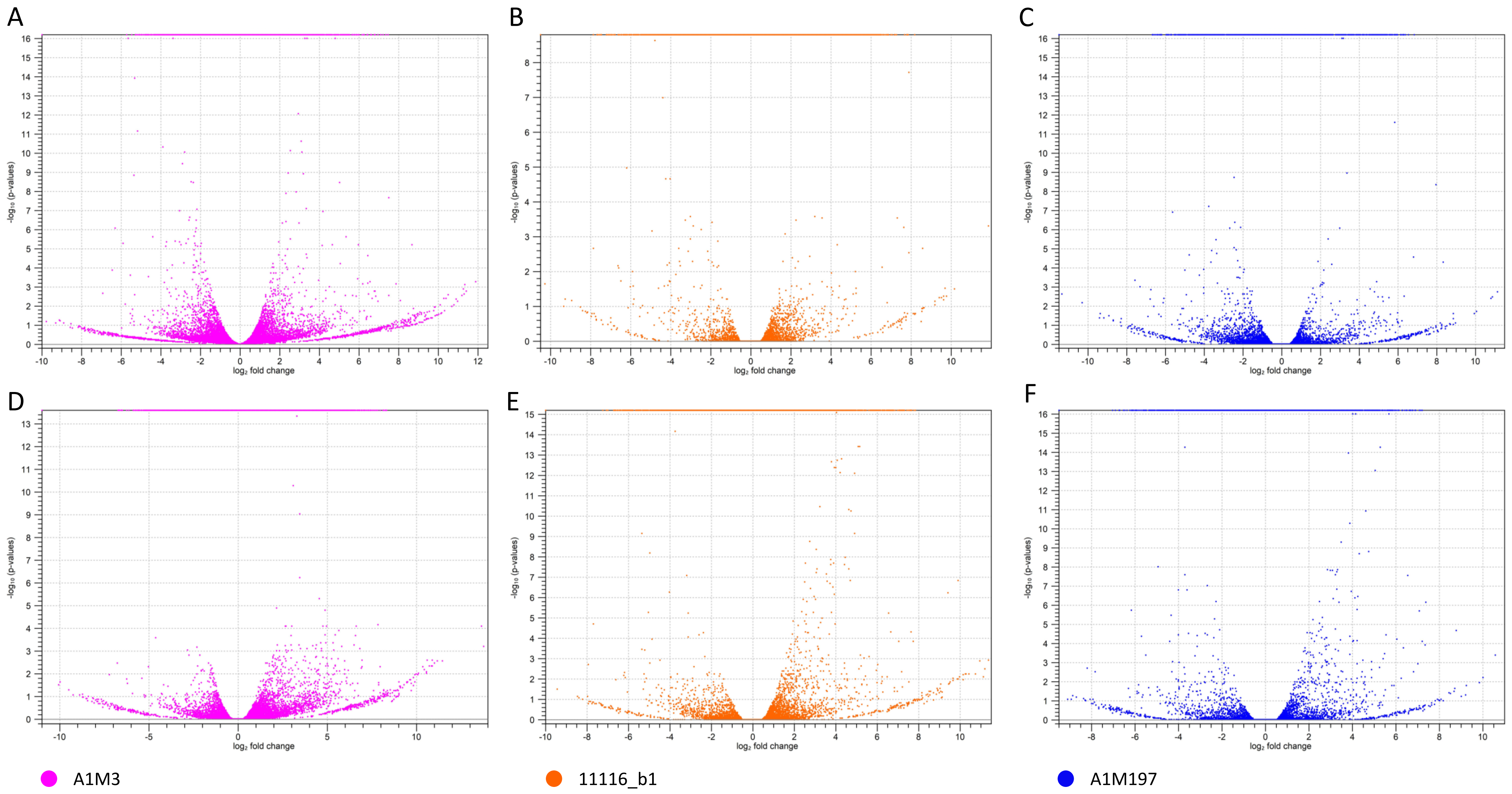

Supplementary Figure S3. Volcano plots showing the differential expression profiles of the datasets from the samples of sweet cherry inoculated with three Pss strains in comparison with mock-treated samples. (A)-(C) Samples taken locally to the inoculation sites. (D)-(F) Samples taken distally to the inoculation sites.

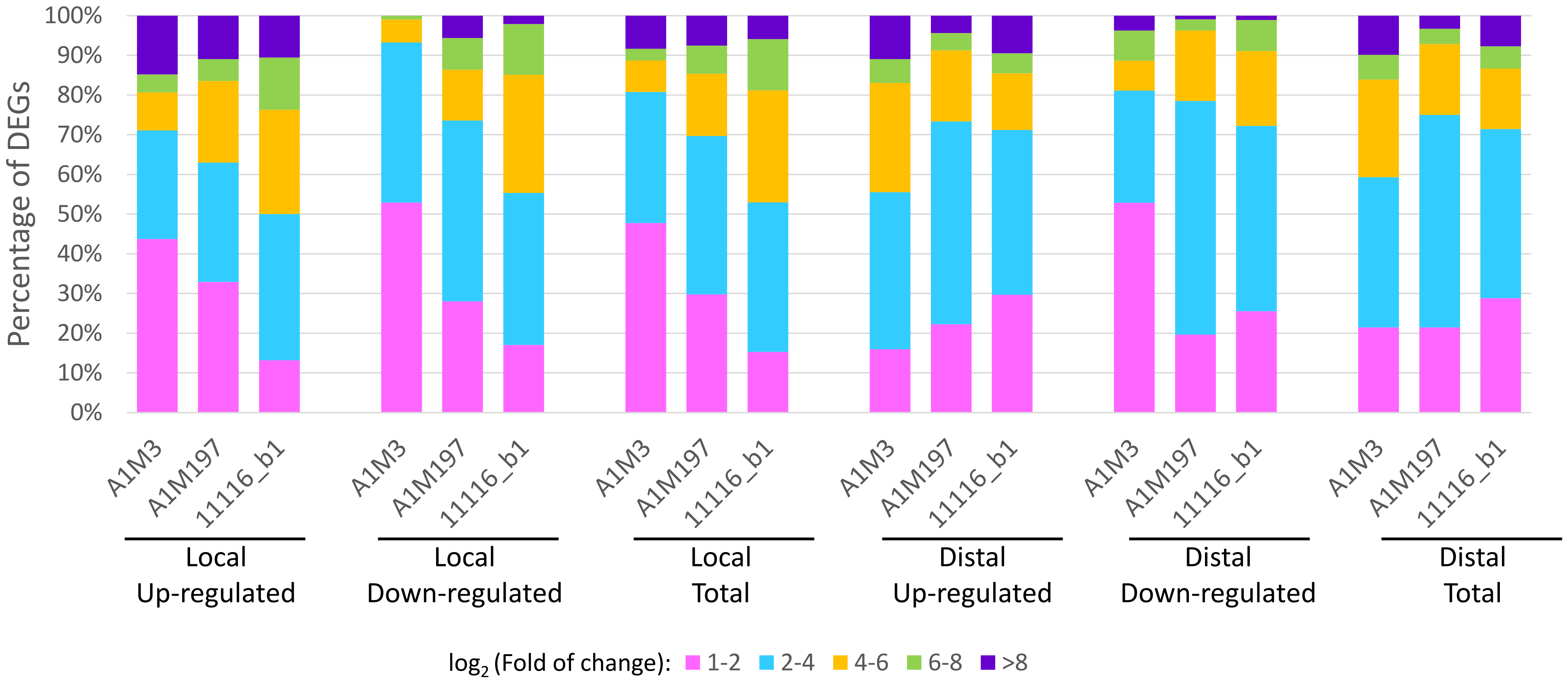

Supplementary Figure S4. Fold change distribution of differentially expressed genes in local and distal samples from sweet cherry inoculated with three Pss strains.

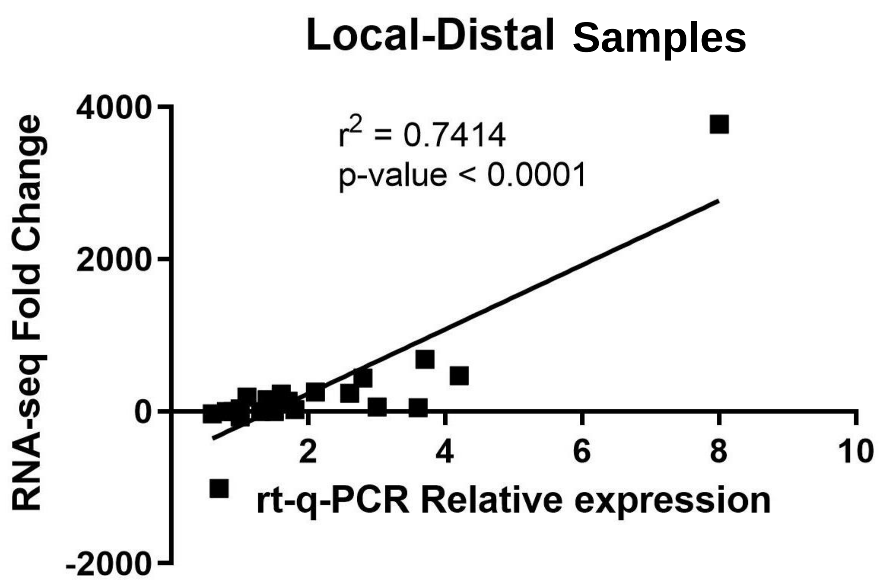

Supplementary Figure S5. Comparison between RNA-seq and qPCR expression data from selected DEGs. Relative expression data of genes from local samples (GolginA-4, E3 ubiquitin ligase PRT6, CBS domain-containing protein, and Actin7) and from distal samples (elF-2- α kinase GCN2, AGD14, GIGANTEA, and Actin7) obtained by qPCR were compared with their RNA-Seq results using simple linear regression. A scatter plot highlights a simple linear regression and shows the coefficient of determination (R^2) and its significance between relative expression obtained by qPCR and its corresponding RNA-seq values.

A B

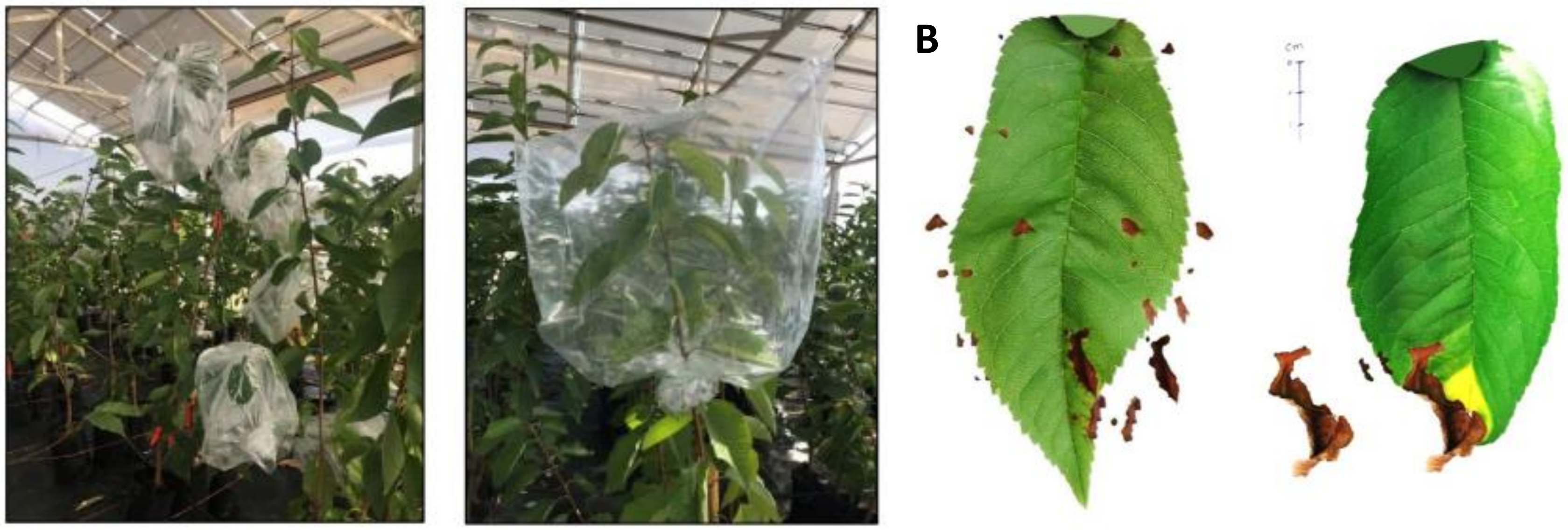

C

Pss Strain	Symptom incidence	Necrotic area
11116_b1	60.0%	1.32%
A1M197	46.7%	1.43%
A1M3	33.3%	0.97%

D

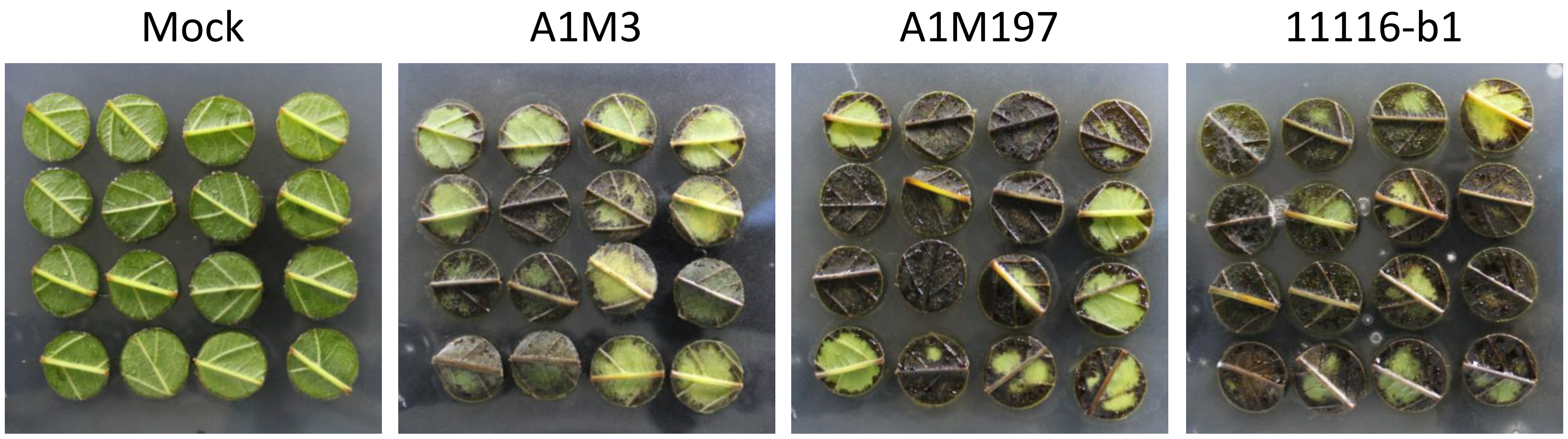

Supplementary Figure S6. Pathogenicity assays of Pss strains in sweet cherry cv. 'Lapins'. (A)-(C) Spray assay. (A) Pss strains (11116_b1, A1M197 and A1M3) were suspended in sterile distilled water and the concentration was adjusted to 10^8 CFU/mL. 50 mL of suspension was sprayed on 30 leaves per plant. The inoculated branches were sealed in plastic bags for two days. (B) Necrotic area on each leaf was measured using ImageJ. (C) Symptoms were evaluated 30 days post inoculation. Symptom incidence, percentage of leaves showing symptoms. Necrotic area, percentage of leaf area that was necrotic. The three Pss strains exhibited varying performance in both parameters. (D) Leaf disk assay adapted from Lienqueo et al. [49]. Freshly picked leaves, approximately 1 to 2 weeks old, were used in the assay. Pss suspension of 2×10^8 CFU/mL (OD_{600} = 0.2) was freshly prepared. Leaves were washed with tap water for 5 min and then superficially disinfected with 70% ethanol for 1 min, followed by three rinses in sterile distilled water. The disinfected leaves were dried with sterile paper towel, and circular leaf disks were obtained using a 17-mm diameter cork borer that had been pre-soaked in the bacterial suspension with 0.01% Silwet L-77 or a mock solution containing 0.8% KCl and 0.01% Silwet L-77. Sixteen leaf disks were evenly placed on each Petri dish containing 10 g/L agar, with the abaxial side facing up. Two Petri dishes were used for each strain or mock inoculation. The plates were then incubated in the dark at 26 °C for 10 days before photos were taken.
