# Supplementary material for: Transcriptome Analysis of Sweet Cherry (Prunus avium L.) Cultivar ‘Lapins’ upon Infection of Pseudomonas syringae pv. syringae
Source: Plants (Basel). 2023 Oct 29;12(21):3718. doi: 10.3390/plants12213718 (PMC10647540; doi:10.3390/plants12213718)
Supplement: Supplementary file 1 [file plants-12-03718-s001.zip › Cui_Supp.Tables.part1.pdf]

Supplementary Table S1. Primers for DNA quantification and DEG validation.

Species	Gene ID	Gene name	Forward Sequence	Reverse Sequence	Amplicon Size (bp)
Pseudomonas syringae* pv. *syringae	(Conserved sequence)	Glutathione-dependent formaldehyde-activating enzyme 1	CACTGCATAACCACGCCTG	AATCCAGACCGTGGAACGC	210
Prunus avium	XM_021955147.1	protein SAR DEFICIENT 1	CATGCTGGGGCAATGTCAAGATCA	CCAGTACCGCATTCAAAGTTGGAG	164
Prunus avium	XM_021979071.1	Golgin subfamily A member 4 (LOC110774526), transcript variant X4	AGCAAGGGTCTTTGGCTTGT	TGTCACGCTGTTGCACCAAT	156
Prunus avium	XM_021969502.1	E3 ubiquitin-protein ligase PRT6 (LOC110766214), transcript variant X2, mRNA	AAGCATGGCGTGGTTACAGT	AAACAGCTCCGCAAACACCA	107
Prunus avium	XM_021962599.1	CBS domain-containing protein CBSCBSPB1-like isoform X2	CGCTGCATACAATGCATGAC	ATAGAGCTTGCGGCCTCATT	157
Prunus avium	XM_021957676.1	eIF-2-alpha kinase GCN2 isoform X2	ATGGAACGACACCATGTGCT	GGAGACATTAAGCGTCGCAA	110
Prunus avium	XM_021962523.1	Protein GIGANTEA isoform X3	TCTGCATTTCCTGCGTGCAA	ACCCAAAGGCTCATGCTGAA	163
Prunus avium	XM_021952030.1	Probable ADP-ribosylation factor GTPase-activating protein AGD14 isoform X2	GCTGTCCAGTCGAAGCTGAT	TGGGAATGTGCTCTACCCTT	159
Prunus avium	XM_021954766.1	peptidyl-prolyl cis-trans isomerase CYP21-1	GACCTGATTCCAATGGCTCACAGT	CAGTGTCCATGCCCTGAATTACCT	105
Prunus avium	XM_021947600.1	Splicing factor 3B subunit 1	TGAGTTTGTTCCAGCGAGGGAATG	GGTTCTGACGTTCCTGCACTTTGA	185
Prunus avium	XM_021976055.1	Actin-7	ATGTGTTGAGGTGGGGTCAGTGAA	AAGATTAGGCAAGGCGAGGATGGA	160

Supplementary Table S2. DNA quantification in *Prunus avium* samples inoculated with different Pss strains. DNA quantification of Pss and *Prunus avium* was performed in three local and distal samples from twigs inoculated with mock control and 11116_b1, A1M197, and A1M3 Pss strains. Mean Cq value (performed in duplicate at least) and DNA concentrations are shown for each sample. Pss DNA presents the ratio between DNA quantification of Pss and *Prunus avium* in each biological replicate. ND, not detected; -, not determined. C+ Pss, purified A1M3 strain DNA; C+ *P. avium*, purified DNA from sweet cherry twig. C-, sterile water.

Sample	Treatment	Replicate	Pss DNA detection		*Prunus avium* DNA detection		Pss DNA (ng/ng)	Mean Pss DNA (ng/ng)
			Cq	DNA (ng/µL)	Cq	DNA (ng/µL)		
Local to the wound	Mock	R1	ND	0	23.3	$5.1x10^{-2}$	0	**$8.4x10^{-7}$**
		R2	35.65	$2.1x10^{-7}$	22.8	$8.3x10^{-2}$	$2.6x10^{-6}$	
		R3	ND	0	23.8	$3.3x10^{-2}$	0	
	11116_b1	R1	33.0	$1.3x10^{-6}$	24.2	$2.3x10^{-2}$	$5.9x10^{-5}$	**$5.7x10^{-5}$**
		R2	33.6	$8.7x10^{-7}$	24.0	$2.8x10^{-2}$	$3.2x10^{-5}$	
		R3	33.5	$9.4x10^{-7}$	24.9	$1.1x10^{-2}$	$8.4x10^{-5}$	
	A1M197	R1	30.8	$6.4x10^{-6}$	23.4	$4.7x10^{-2}$	$1.4x10^{-4}$	**$4.0x10^{-4}$**
		R2	32.3	$2.3x10^{-6}$	23.4	$4.8x10^{-2}$	$4.7x10^{-5}$	
		R3	28.8	$2.6x10^{-5}$	24.1	$2.5x10^{-2}$	$1,1x10^{-3}$	
	A1M3	R1	35.7	$2.0x10^{-7}$	23.3	$4.9x10^{-2}$	$4.1x10^{-6}$	**$8.8x10^{-6}$**
		R2	33.9	$7.5x10^{-7}$	22.7	$8.9x10^{-2}$	$8.4x10^{-6}$	
		R3	33.3	$1.1x10^{-6}$	22.8	$7.9x10^{-2}$	$1.4x10^{-5}$	
Distal to the wound	Mock	R1	ND	0	30.7	$5.4x10^{-5}$	0	**0**
		R2	ND	0	30.6	$6.1x10^{-5}$	0	
		R3	ND	0	29.6	$1.6x10^{-4}$	0	
	11116_b1	R1	ND	0	28.2	$5.8x10^{-4}$	0	**0**
		R2	ND	0	29.5	$1.7x10^{-4}$	0	
		R3	ND	0	29.9	$1.1x10^{-4}$	0	
	A1M197	R1	35.6	$2.2x10^{-7}$	28.4	$4.8x10^{-4}$	$4.7x10^{-4}$	**$1.5x10^{-4}$**
		R2	ND	0	29.5	$1.7x10^{-4}$	0	
		R3	ND	0	28.2	$5.8x10^{-4}$	0	
	A1M3	R1	ND	0	27.8	$8.3x10^{-4}$	0	**$4.7x10^{-1}$**
		R2	ND	0	29.2	$2.2x10^{-4}$	0	
		R3	24.8	$4.2x10^{-4}$	28.9	$3.1x10^{-4}$	1.4	
	**C+ Pss**		15.2	$3.3x10^{-1}$	-	-	-	**-**
	**C+ *P. avium***		-	-	28.7	$3.5x10^{-4}$	-	**-**
	**C-**		ND	0	ND	0	-	**-**
